# A comprehensive data set of lake surface water temperature over the Tibetan Plateau derived from MODIS LST products 2001–2015

**DOI:** 10.1038/sdata.2017.95

**Published:** 2017-07-25

**Authors:** Wei Wan, Huan Li, Hongjie Xie, Yang Hong, Di Long, Limin Zhao, Zhongying Han, Yaokui Cui, Baojian Liu, Cunguang Wang, Wenting Yang

**Affiliations:** 1 State Key Laboratory of Hydroscience and Engineering, Tsinghua University, Beijing 100084, China; 2 Department of Hydraulic Engineering, Tsinghua University, Beijing 100084, China; 3 Department of Geological Sciences, University of Texas at San Antonio, San Antonio, Texas 78249, USA; 4 Department of Civil Engineering and Environmental Science, University of Oklahoma, Norman, Oklahoma 73019, USA; 5 Institute of Remote Sensing and Digital Earth, Chinese Academy of Sciences, Beijing 100101, China; 6 The Center for National Spaceborne Demonstration, Beijing 100101, China; 7 School of Earth and Space Sciences, Peking University, Beijing 100871, China

**Keywords:** Hydrology, Climate change, Limnology

## Abstract

Lake surface water temperature (LSWT) is sensitive to long-term changes in thermal structure of lakes and regional air temperature. In the context of global climate change, recent studies showed a significant warming trend of LSWT based on investigating 291 lakes (71% are large lakes, ≥50 km^2^ each) globally. However, further efforts are needed to examine variation in LSWT at finer regional spatial and temporal scales. The Tibetan Plateau (TP), known as ‘the Roof of the World’ and ‘Asia’s water towers’, exerts large influences on and is sensitive to regional and even global climates. Aiming to examine detailed changing patterns and potential driven mechanisms for temperature variations of lakes across the TP region, this paper presents the first comprehensive data set of 15-year (2001–2015) nighttime and daytime LSWT for 374 lakes (≥10 km^2^ each), using MODIS (Moderate Resolution Imaging Spectroradiometer) Land Surface Temperature (LST) products as well as four lake boundary shapefiles (i.e., 2002, 2005, 2009, and 2014) derived from Landsat/CBERS/GaoFen-1 satellite images. The data set itself reveals significant information on LSWT and its changes over the TP and is an indispensable variable for numerous applications related to climate change, water budget analysis (particularly lake evaporation), water storage changes, glacier melting and permafrost degradation, etc.

## Background & Summary

Lakes, particularly alpine lakes, act as sentinels by producing and storing signals that reflect the influence of climate change on terrestrial and aquatic ecosystems^1^. Changes in lakes reflect the changes of physical, chemical and biological processes in the surrounding catchment due to climate forcing^2^. Lake surface water temperature (LSWT), as being directly related to and sensitive to long-term changes in thermal structure of lakes and regional climate, is an important indicator to understand changes in lake state^2,3^ as well as surrounding basins and regional climate change. Recent studies on large lakes globally showed an overall marked warming trend that is clearly a response to the current warming climate^4,5^, whilst several studies on regional scale showed various degrees of responses^6,7,8^.

The Tibetan Plateau (TP), a vast highland area in Central Asia, also known as ‘the Roof of the World’ and ‘Asia’s water towers’^9^, exerts a large influence on and is sensitive to regional and global climate through thermal and mechanical forcing mechanisms^10^. The TP consists of more than 1,100 large alpine lakes (>1 km^2^ each), with the total area exceeding 46,527 km^2^ (ref. 11). Recent studies have examined the status and change in lake area^12,13^, water level^14,15^, and water volume^16,17^ across the TP. The status and change in LSWT, however, still remain unclear, although the LSWT of over 50 large lakes has been previously reported^8,18^.

**Figure 1:**
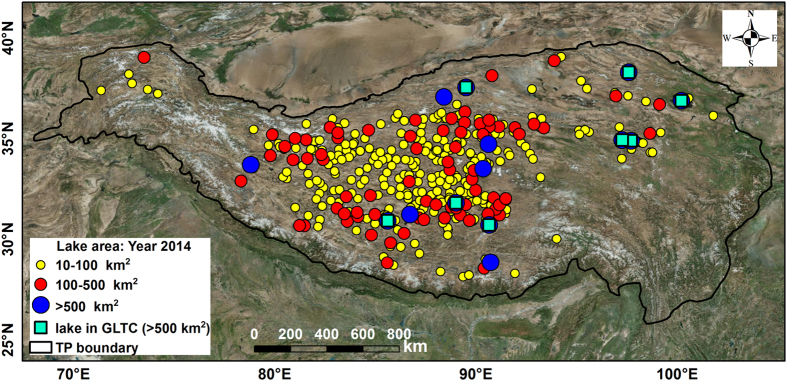
Map of lakes included in the generated data set. The boundary of the TP is defined as above the elevation of 2,500 m using NASA Shuttle Radar Topographic Mission (SRTM) 90 m Digital Elevation Models (DEM) Database v4.1^11^. (GLTC: Global Lake Temperature Collaboration).

**Figure 2:**
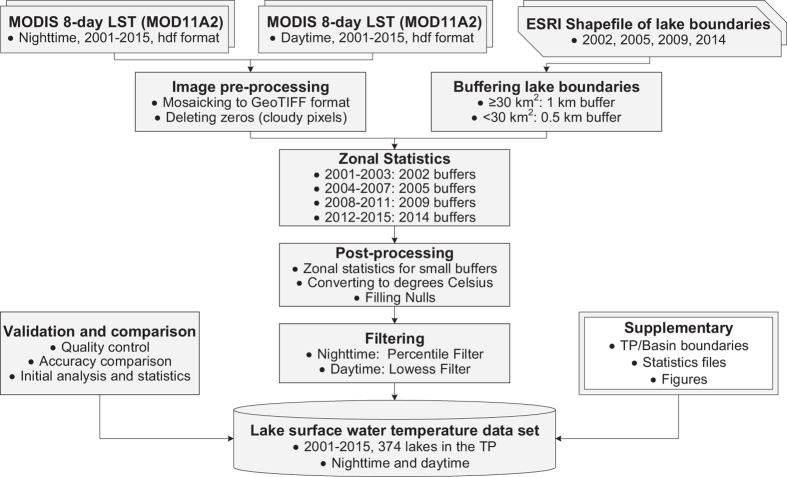


There are several different global-scale lake temperature data products which are publically accessible, e.g., the data set of ATSR Reprocessing for Climate: LSWT & Ice Cover (ARC-Lake, http://www.laketemp.net/, version 3 from 1995–2012 was released in year 2014)^19^, and the data set of lake temperature (1985–2009) created by the Global Lake Temperature Collaboration (GLTC, www.laketemperature.org) in 2015 (ref. 20). There are only a few regional-scale LSWT products that are publically available, e.g., the LSWT products for European Alpine lakes (1989–2013) using Advanced Very High Resolution Radiometer (AVHRR) data^21^. Furthermore, except for ARC-Lake, the above-mentioned existing data sets only included nighttime temperatures. This is insufficient to represent large diurnal variations experienced in some lakes, particularly large in small systems^22,23^. To date, there is no comprehensive data set on LSWT across the TP, especially no open-access data sets derived from satellite image; even the ARC-Lake and GLTC include only large lakes (i.e., ≥ 50 km^2^ each) within the TP region. In this paper, we present the first 15-year (2001–2015) comprehensive data set of nighttime and daytime LSWT from 374 lakes with areas greater than 10 km^2^ each (Figure 1). We used MODIS (Moderate Resolution Imaging Spectroradiometer) Land Surface Temperature (LST) 8-day composite products (MOD11A2, level 3), as well as four lake boundary files in ArcGIS format (i.e., 2002, 2005, 2009, and 2014) derived from Landsat/CBERS/GaoFen-1 satellite images, as the main data sources. The flowchart for producing and validating this data set is shown in Figure 2. This newly generated data set could be valuable in addressing scientific questions associated with regional climate change, water budget analysis (particularly lake evaporation), surface water storage changes, glacier melting and permafrost degradation, etc.

## Methods

### MODIS LST product pre-processing

MODIS/Terra LST level 3, 8-day average, per-pixel data of 1-km nominal spatial resolution (MOD11A2, version 6) were obtained from NASA’s Earth Observing System Data and Information System (EOSDIS, https://earthdata.nasa.gov). MOD11A2 is the averaged LSTs of daily MOD11A1 products over 8 days, including 46 8-day samples per year. Zhang *et al.*^8^ showed a comprehensive explanation of the reliability of choosing MOD11A2 as a basic MODIS data source in addition to others (e.g., MYD11A2). Day- and night-time LST layers (overpass time ~10.30 am and ~10.30 pm of local time, respectively) were extracted from MOD11A2. The LST tiles were resampled to a 1-km pixel size and reprojected onto a Geographic (GEO) projection using the MODIS Reprojection Tool (https://lpdaac.usgs.gov/tools/modis_reprojection_tool) from the original sinusoidal projection. The original tiles were then mosaicked and converted to a GeoTIFF format. Cloud-contaminated pixels in the raw MOD11A2 data were replaced with Nulls.

### Lake boundary data processing

A previous study presented lake boundary data sets of the TP region for the 1960s, 2005, and 2014 (ref. 11). To match the time period of the Ice, Clouds, and Land Elevation Satellite (ICESat) and CryoSat-2 for monitoring lake level changes^14,15^, in this study, we further produced two more lake boundaries (i.e., 2002 and 2009) for lakes with area greater than 10 km^2^, using Landsat TM and ETM+ images and following the same procedure described in Wan *et al.*^11^. Considering the time periods of MODIS data in this study (i.e., 2001–2015), lake boundary shapefiles for 2002, 2005, 2009 and 2014 were used together as references for LSWT extraction. To reduce potential errors due to the fluctuation of land-water interfaces or the inconsistency of year-by-year water boundaries attributed to the geometric distortion of the GaoFen-1 images, and to reserve as many lakes as possible for LSWT extraction, a 1-km buffer zone offshore for lakes with area ≥ 30 km^2^ and a 0.5-km buffer zone offshore for lakes with area 10–30 km^2^ were generated, to exclude pixels along shorelines. Overlapped lakes appearing in all the four buffer shapefiles were selected and saved. Following the above steps, we finally obtained four lake buffer shapefiles, corresponding to the four lake boundary shapefiles, for a total of 374 lakes. The four buffer shapefiles were then used as base files (masks) for LSWT extraction from the MOD11A2 files.

### LSWT deriving

A stepwise processing method was used to derive the final LSWT products and is described in detail as follows:

#### 1) Zonal statistics

Lakes over the TP were either expanding or shrinking over the past few decades^24,25,26,27^. In order to ensure accurate lake boundaries, for a specific year, we used the buffer boundary shapefile of lakes that was temporally close to the pre-processed MODIS LST data to represent the buffer zone of lakes. As shown in Figure 2, the 2002, 2005, 2009, and 2014 buffer shapefiles were used for masking the 2001–2003, 2004–2007, 2008–2011, and 2012–2015 MODIS data, respectively. For each year, the mean and standard deviation (STD) values of each lake were summarized within the zone of each buffer shape of the lake.

#### 2) Post-processing

The majority of the lakes met the above-mentioned rules of zonal statistics. However, there were some lakes whose buffer areas were smaller than one pixel of the MODIS LST data; as a result, these lakes received no mean and STD values of temperature. To resolve this problem, we created a feature class containing points generated from the representative locations of those small buffers. This point-layer was then used to execute the zonal statistics to obtain temperature values for these lakes. After combining the above two statistics for means and STDs together, we converted the raw pixel values of the mean LST into degrees Celsius (°C) using a simple equation *P*×0.02–273.15, where *p* denotes the mean LST value of each lake. For a cloudy day with Null values for some lakes, the values were filled through interpolations using the temperature values before and after the present day (i.e., 8-day since it is the 8-day product).

#### 3) Filtering

**Figure 3:**
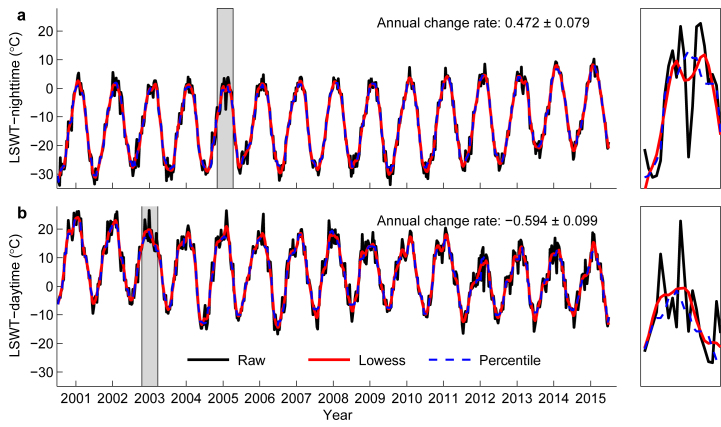
Performances of the two filtering methods. (**a**) Yake Co (34.70°N, 87.19°E) for nighttime time series; (**b**) Xiaokusai Lake (36.08°N, 92.80°E) for daytime time series. Black lines denote the raw LSWT data with Nulls being filled for the period 2001–2015; Red solid lines and blue dash lines denote the Lowess Filter and Percentile Filter, respectively.

For the filled data in 2), due to the limitations of methods for removing cloudy data in the MODIS LST products^28^, there were still cloud-contaminated outliers in the data set, particularly for the nighttime data in summer. Previous studies applied different approaches to take into account this issue, such as using air temperature as a reference^8^, the MODIS quality control data sets (QC SDS)^29^, and filtering^18^. Here, we tested the effectiveness of several filters for both nighttime and daytime data and eventually chose two types of filters, i.e., the Percentile Filter and the Lowess Filter^30^. Figure 3 shows the performance of the two filtering methods on nighttime (**a**) and daytime (**b**) time series for two typical lakes: one is Yake Co (34.70°N, 87.19°E, NAME_ID=334) with the highest annual increasing rate (0.472±0.079 °C/year) for nighttime LSWT, and the other is Xiaokusai Lake (36.08°N, 92.80°E, NAME_ID=316) with the highest annual decreasing rate (−0.594±0.099 °C/year) for daytime LSWT. Note that for nighttime data, the Percentile Filter performed better than the Lowess Filter since it can smooth summer outliers in a more effective way. For daytime data, the Lowess Filter performed better since it can reserve more details while smoothing. For Percentile Filter, a fixed span value (i.e., span=5) was used for all data series, whilst for Lowess Filter, the fixed value was span=8.

## Data Records

The overall information for the 374 investigated lakes is shown in the ‘LAKE_INFO.csv’ file, i.e., name, latitude/longitude, basin being located in, etc. The basins in which the lakes are located are numbered in alphabetical order: 1-AmuDarya, 2-Brahmaputra, 3-Hexi, 4-Indus, 5-Inner TP, 6-Qaidam, 7-Salween, 8-Tarim, 9-Yangtze, and 10-Yellow (Two basins, i.e., Ganges and Mekong, with no target lakes in them, are numbered with −999.). The Inner TP basin was subdivided into 6 small basins ranging from 51-InnerA to 56-InnerF. The data set is available in three folders. The ‘nighttime’ and ‘daytime’ folders, organized in the same way, contains four subfolders, ‘raw’, ‘filled’, ‘filter’, and ‘annual_monthly_mean’, respectively. Data organization and description are presented in Table 1. All the files containing the actual values can be linked to each other via the NAME_ID column. The 2005 and 2014 lake boundary data in the ‘shapefile’ folder used in this study were published in Wan *et al.*^11^. The 2002 and 2009 lake boundary data as well as all the LSWT data are first published here. The data set can be accessed at (Data Citation 1).

**Figure 4:**
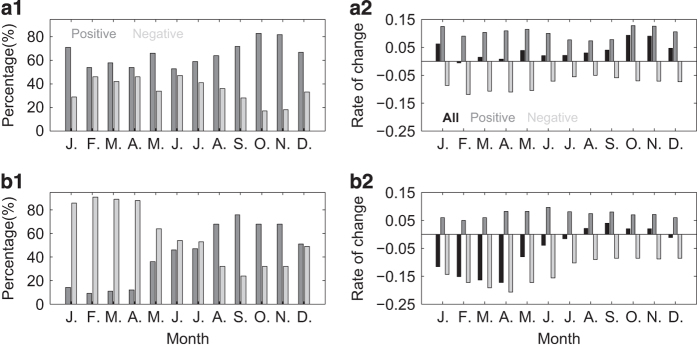
Monthly statistics for the lake surface water temperature (LSWT) changes from this data set (2001–2015): Change percentage (**a1**,**b1**) and rate (**a2**,**b2**) of positive lakes (red), negative lakes (blue), and all lakes (black), in nighttime (**a1**,**a2**) and daytime (**b1**,**b2**), respectively.

For each lake in the new data set, the annual rate of change in temperature was derived using annual mean LSWT(°C) regressed against data acquisition year using the regression model described in Zhang *et al.*^8^. The monthly mean LSWT (°C) was calculated by averaging the 8-day samples whose DOYs were exactly within the range of a specific month. An overall monthly statistic for the variation in change percentage and rate of lakes in this data set is shown in Fig. 4. Figure 4 (a1) show positive or negative change percentages of the LSWT, for nighttime and daytime, respectively. Note that the percentages of positive lakes in September (72%, 76%), October (83%, 68%), and November (82%, 68%) are high for both nighttime and daytime. January for nighttime data also shows a high percentage (71%) for positive lakes. For daytime data, >85% lakes show decreasing rate from January to April. Figure 4 (a2) show change rates of LSWT for all lakes, positive lakes and negative lakes, in nighttime and daytime, respectively. Note that the change rates show two peaks for both positive and negative lakes in nighttime, whereas in daytime, there is only one peak at around June for positive lakes and April for negative lakes.

## Technical Validation

### Quality control and assurance of the data set

We executed quality control for producing both the lake/buffer boundary data and the LSWT data. To produce the 2002 and 2009 lake boundary shapefiles, consistent rules for producing the previous data set published in Wan *et al.*^11^ were used by the same group and through the same techniques. The shapefiles for the lake and buffer boundaries were overlaid and examined in ESRI ArcGIS software carefully to ensure the consistency of the geographic locations and the shape. For each lake, Lake name and ID were examined to avoid any mismatching problem. To reduce erroneous values, the initial results were cross-checked lake by lake by three co-authors of this paper (Wei Wan, Huan Li, and Zhongying Han) to ensure that there were no erroneous lakes.

Each step was strictly controlled for producing the LSWT data set. After the assembly of the data set was completed, for the raw, filled, and filtered data sets, we examined the distribution of lake temperatures and the monthly mean values using a series of plots. These plots allowed us to identify outliers and unusual trends. Attention was paid to examine those lakes with abnormal temperature variations to ensure that we extracted LST values from the water surface rather than the land surface or mixed water and land surfaces. We also compared the distributions and trends of the change rates using maps and data from previous publications. Primary conclusions are shown in the final section of this paper.

Preliminary and interesting discoveries are found from the data set, e.g., for daytime data, in Gansenquan Lake (37.46°N, 92.77°E, NAME_ID=105) in Qaidam basin, a very high temperature in summer was observed (monthly mean exceeding 40 °C for June, July, and August). This was consistent with the maximum air temperature in this basin which can reach a high range of 40 °C −55 °C^31^. Another phenomenon was that the majority of lakes in the Brahmaputra basin exhibited abnormal seasonal cycles, in particular, Chumba Yumco (28.23°N, 89.64°E, NAME_ID=59) and Nariyong Co (28.30°N, 91.95°E, NAME_ID=203). Driving mechanisms contributing to this phenomenon should be further investigated.

### Comparisons with other data sets and *in situ* measurements

**Figure 5:**
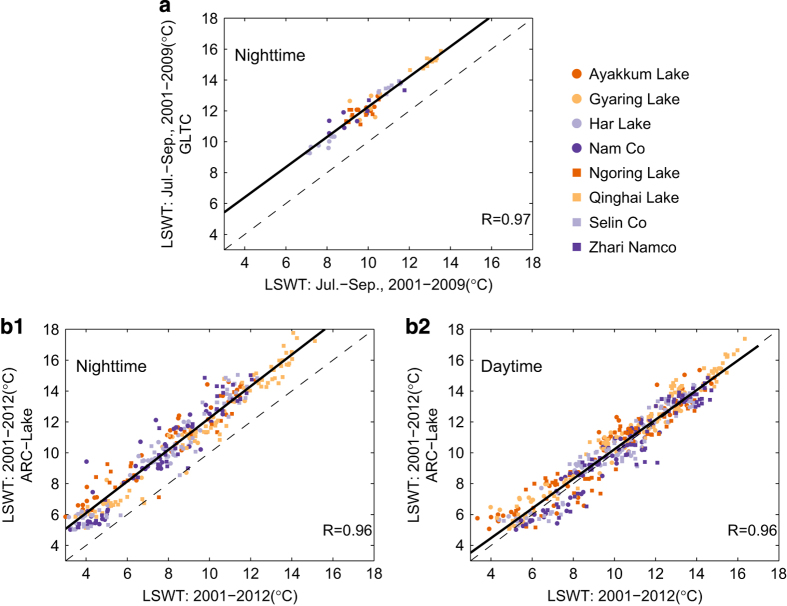
Correlations between the generated data set in this study and the other two data sets for eight same lakes during the corresponding overlap periods. (**a**) the GLCT; (**b1**) the Nighttime data set of the ARC-Lake, and (**b2**) the Daytime data set of the ARC-Lake.

After validating the accuracy of the extraction process for the developed data set, we further compared our data set to the two global data sets, i.e., the GLTC and ARC-Lake^20^. The GLTC data set assembled a global database of summer lake temperatures for 291 lakes collected *in situ* and/or by satellites for the period 1985–2009. There were eight lakes in both the GLTC data set and the new data set presented here that have data in the overlap period of 2001–2009 (Figure 1). These eight lakes are Ayakkum Lake (37.55°N, 89.42°E), Gyaring Lake (34.93°N, 97.26°E), Har Lake (38.29°N, 97.59°E), Nam Co (30.74°N, 90.60°E), Ngoring Lake (34.90°N, 97.70°E), Qinghai Lake (36.88°N, 100.20°E), Selin Co (31.80°N, 88.99°E), and Zhari Namco (30.93°N, 85.62°E). The surface temperatures of the eight lakes from the GLTC data set were collected by satellite data from either the Advanced Very High Resolution Radiometer (AVHRR) or the Along Track Scanning Radiometer (ATSR-1, ATSR-2, AATSR). The summer mean temperatures were calculated for a 3-month period from 1 July to 30 September. The final temperature presented in the GLTC data set was the bulk water temperatures calculated using satellite-derived surface temperature and sensor specific correction factors described in Sharma *et al.*^20^. The correlations between the two data sets for the eight lakes are shown in Figure 5(a). Note that the two data sets show pretty high agreement (*R*=0.97) with an average bias of ~2.5 °C, with GLTC values higher than MODIS values. The bias could be attributed to two factors, i.e., one is the systematic deviation between the surface temperature (MODIS) and bulk temperature (GLTC) (i.e., surface temperature is~0.46 °C smaller than the bulk temperature as reported in Hook *et al.*^32^), and the other is the systematic deviation between the different satellite sensors.

The ARC-Lake data set (version 3) included ATSR-2/AATSR-based lake surface temperatures for globally-distributed 1,628 target water bodies. The LSWTs of the above-mentioned eight lakes were used to compare between the ARC-Lake data set and the data set generated in this study. The two data sets used different strategies to deal with the LSWTs in frozen periods, i.e., the ARC-Lake substituted the frozen LSWTs with 0 °C based on climatologic approach, whilst the data set in this study used the original ice surface temperatures from MODIS. Therefore, only LSWTs higher than 5 °C were used for comparison (the additional 5 °C was to eliminate deviations generated from the climatologic algorithms). Figure 5(b1 show correlations between the monthly mean LSWTs of the two data sets for nighttime and daytime, for the overlap period from Jan. 2001-Mar. 2012, respectively. Note that the two data sets show pretty high agreements (*R*=0.96 for both), with an average bias of ~2.2 °C for nighttime data, however. Since both of the two data sets measured the lake surface temperatures by satellites, the bias from nighttime could be attributed to the systematic deviation between the different satellite sensors.

Satellite measurements of the LWSTs are biased due to characteristics such as weather-dependent, instantaneity in time, and methodology-dependent. It is crucial that the satellite data be validated against *in situ* data. The absolute differences between MODIS LST products and *in situ* measurements have been reported to be within the range of 0.8–1.9 K^33,34,35^. Several studies have compared the two for lakes over the TP region, which showed biases (LSWT versus *in situ*) of −1.74 °C^18^ and −1.4 °C^8^. Here, due to a lack of field observations in the TP, we did not directly compare our data set with *in situ* measurements, instead, in the following section, we compared the change rates calculated from our data set with those from the above-mentioned publications which had already validated using *in situ* mesurements^8,18^.

### Evaluation of change rates from different data sources

**Figure 6:**
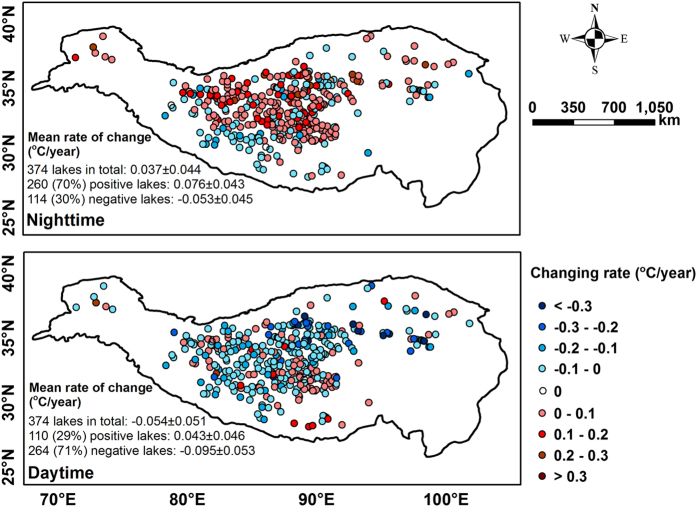
Change rates of LSWT for the observed 374 lakes in both nighttime (upper panel) and daytime (bottom panel) over 2001–2015. Blue and red solid circles represent positive and negative change rates, respectively. The values on lower left of each panel are the mean rate of change (°C/year)±the s.e. for all, positive, negative lakes, respectively.

**Figure 7:**
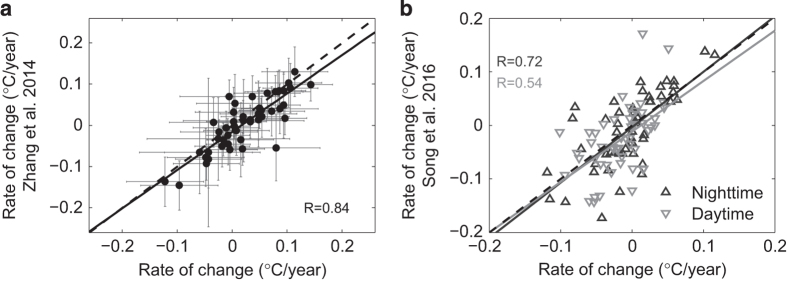
Correlations of change rates of LSWT derived from the generated data set and that from the other two data sets. (**a**) Zhang *et al.*^8^; (**b**) Song *et al.*^18^. Error bars in (**a**) is the s.e. for trend estimation calculated using method described in Zhang *et al.*^8^.

Using the data set produced in this study, Figure 6 shows change rates of LSWT for the observed 374 lakes in both nighttime and daytime during 2001–2015. Note that long-term trends in LSWT showed opposite patterns in nighttime and daytime, with overall trends of 0.037±0.044 °C/year in nighttime and −0.054±0.051 °C/year in daytime. For nighttime data, 70% of the lakes showed increasing rates (mean 0.076±0.043), while only 30% showed decreasing rates (mean −0.053±0.045). For daytime data, the majority of lakes (71%) showed decreasing rates with a pretty low mean value of −0.095±0.053. 19%/21% of the lakes show consistent trend (warming/cooling) across day/night. This is generally consistent with results from 56 large lakes investigated in Song *et al.*^18^. We further compared our change rates with results published in the above-mentioned two studies, i.e., the 2001–2012 data for nighttime generated by Zhang *et al.*^8^ and the 2001–2015 data for both nighttime and daytime generated by Song *et al.*^18^. Both of the two data records used MOD11A2 as the original data source and compared with *in situ* measurements of both surface temperature and air temperature. Figure 7(a) shows the correlations between the change rates of LSWT derived from the generated new data set and those from Zhang *et al.*^8^. The two data sets showed good agreement with correlation coefficient of *R*=0.84. Note that our data are slightly higher than Zhang’s in general. This is reasonable since, for estimating LSWT, we averaged values of all pixels within the lake buffer boundaries whereas Zhang *et al.*^8^ used the averages from a 3×3 pixel window in the center of each lake. That method (Zhang *et al.*^8^), however, restricted the qualified lakes to only 56, whereas our method of utilizing every available non-cloud pixel value of each lake allowed 374 lakes qualified for value extraction, resulting in a more comprehensive and detailed picture of variations in LSWT across the TP. Figure 7(b) shows the correlations of the change rates of LSWT between our new data set and the data set generated by Song *et al.,*^18^ for both nighttime (*R*=0.72) and daytime (*R*=0.54). The reasons for this discrepancy between the two are perhaps attributed to different methods used for deriving lake boundaries and filtering. Lake boundaries from the Song’s data were static which were generated using the water index based on the NIR/Green ratio of Landsat TM images mainly from the year 2008, whilst we used four lake boundary shapefiles manually extracted from satellite images to account for lake water fluctuations during the 15-years period. The relatively lower consistency between the two for daytime is mainly attributed to the effects of solar irradiance from inconsistent lake boundaries inherited in the Song *et al.*^18^ method. In other words, this shows an accuracy difference between the two data sets, depending on lake size, since the smaller lakes or those that experienced greater changes, the LSWT would deviate more from a static lake boundary.

## Additional Information

**How to cite this article:** Wan, W. *et al.* A comprehensive data set of lake surface water temperature over the Tibetan Plateau derived from MODIS LST products 2001–2015. *Sci. Data* 4:170095 doi: 10.1038/sdata.2017.95 (2017).

**Publisher’s note:** Springer Nature remains neutral with regard to jurisdictional claims in published maps and institutional affiliations.

## Supplementary Material



## Figures and Tables

**Table 1:** Data organizations and descriptions for the generated data set.

**Folder**	**Subfolder**	**File name**	**Data label**	**Description**
shapefile	boundary_shp		ESRI shapefiles for boundaries of the TP region as well as the 12 great basins and 6 subbasins inside the great basin Inner TP; The Basin_IDs are generally consistent with those in the following ‘lake_shp’ and ‘buffer_shp’ shapefiles, except that there are two basins (i.e., Ganges and Mekong) with no target lakes in them are labelled with −999.	
	lake_shp	Lake data set for the TP region from 2002, 2005, 2009, and 2014, in ESRI shapefile format	ID	Lake code in the industry standard^36^
			SHAPE	Feature type of the lake object
			NAME_CH	Chinese name of the lake
			NAME_EN	English name of the lake
			LAT_NORTH	North latitude of the geometric centre of the lake polygon in decimal degree
			LONG_EAST	East longitude of the geometric centre of the lake polygon in decimal degree
			PERIMETER	Perimeter of the lake polygon in kilometre
			WATER_S	Area of the lake’s water surface in square kilometre
			BASIN	The name of the basin where the lake is located in
			BUFF_DIST	Buffer distance offshore for the lake. −1 indicates 1 km and −0.5 indicates 0.5 km
			NAME_ID	Digital IDs for the 374 lakes. The same meaning for all the files in the new data set
			BASIN_ID	Digital IDs for the basins used in the new data set
	buffer_shp		Buffer shapefiles for the 2002, 2005, 2009, and 2014 lake data set in the 'lake_shp' folder, in ESRI shapefile format. Data labels and descriptions could be refered to the ‘lake_shp’ shapefiles.	
			nighttime		LSWT_N_raw.txt	MEAN_001~MEAN_361	**N1**: Raw LSWT(°C) derived from nighttime MODIS 8-day LST products (MOD11A2)
		LSWT_N_filled.txt		**N2**: Raw LSWT(°C) with Nulls in **N1** being filled		
		LSWT_N_filter.txt		**N3**: Filtered LSWT(°C) in **N2** using Percentile Filter		
		LSWT_N_monthly_mean.txt	MEAN_001~MEAN_012	Nighttime monthly mean LSWT(°C) from Jan. to Dec.		
		LSWT_N_annual_mean.txt	MEAN_2001~MEAN_2015	Nighttime annual mean LSWT(°C)		
			RATE	Nighttime annual change rate		
			SE	Standard error for nighttime change rate		
			daytime		LSWT_D_raw.txt	MEAN_001~MEAN_361	**D1**: Raw LSWT(°C) derived from daytime MODIS 8-day LST products (MOD11A2)
		LSWT_D_filled.txt		**D2**: Raw LSWT(°C) with Nulls in **D1** being filled		
		LSWT_D_filter.txt		**D3**: Filtered LSWT(°C) in **D2** using Percentile Filter		
		LSWT_D_monthly_mean.txt	MEAN_001~MEAN_012	Daytime monthly mean LSWT(°C) from Jan. to Dec.		
		LSWT_D_annual_mean.txt	MEAN_2001~MEAN_2015	Daytime annual mean LSWT(°C)		
			RATE	Daytime annual change rate		
			SE	Standard error for daytime change rate		
			figures		*yyyy*_N_filter.png	Plots of the filtered nighttime LSWT(°C) for the 374 lakes	
		*yyyy*_D_filter.png	Plots of the filtered daytime LSWT(°C) for the 374 lakes			
		*yyyy*_N_monthly_mean.png	Plots of the nighttime monthly mean LSWT(°C) for the 374 lakes			
		*yyyy*_D_monthly_mean.png	Plots of the daytime monthly mean LSWT(°C) for the 374 lakes			
